# A systematic review of physical activity guidelines for adults with cardio-respiratory diseases: Stepping towards evidence-based recommendations

**DOI:** 10.1177/02692155261441158

**Published:** 2026-04-24

**Authors:** Eda Tonga, Thomas Yates, Hannah Worboys, Sally J Singh, Pip Divall, G Andre Ng, Rachael A Evans

**Affiliations:** 1 Department of Global, Lifestyle and Metabolic Health, School of Medical Sciences, Diabetes Research Centre, 4488 University of Leicester, Leicester, UK; 2 National Institute for Health Research, Leicester Biomedical Research Centre, Leicester, UK; 3 Leicester British Heart Foundation Centre of Research Excellence, University of Leicester, Leicester, UK; 4 Biostatistics Research Group, Department of Population Health Sciences, College of Life Sciences, 4488 University of Leicester, Leicester, UK; 5 Centre of Exercise and Rehabilitation Sciences, NIHR Leicester Biomedical Research Centre, University Hospitals of Leicester, Leicester, UK; 6 Institute for Lung Health, Department of Respiratory Sciences, 4488 University of Leicester, Leicester, UK; 7 4490 University Hospitals of Leicester NHS Trust, Leicester, UK; 8 Department of Cardiovascular Sciences, 4488 University of Leicester, Glenfield Hospital, Leicester, UK

**Keywords:** COPD, asthma, heart failure, physical activity, guidelines

## Abstract

**Objectives:**

Physical activity benefits for adults with cardio-respiratory diseases are well established, and evidence-based recommendations are essential for healthcare professionals. This study systematically reviewed existing recommendations on physical activity for adults with cardio-respiratory diseases, specifically chronic obstructive pulmonary disease, asthma, and heart failure, focusing on the frequency, intensity, time and type (FITT).

**Data Sources:**

We searched OVID MEDLINE, EMBASE, CINAHL, and grey literature for guidelines and related documents. The comprehensive search was conducted in July 2025 and subsequently updated through March 2026. Two authors independently screened guidelines, extracted FITT components, and documented disease-specific precautions. Disagreements were resolved with a third author. The AGREE II instrument assessed methodological quality for identified CPGs. Recommendations were categorised based on the FITT framework.

**Results:**

We included 29 guidelines, of which 14 were classified as Clinical Practice Guideline and assessed with AGREE II. Among the 14 guidelines, 7 demonstrated high quality, 6 were moderate, and 1 was low quality. Most guidelines recommended at least 150 minutes of moderate aerobic activity per week. Adaptive recommendations primarily addressed exacerbations and symptom management.

**Conclusion:**

While aerobic physical activity was consistently recommended, disease-specific guidance and adherence to FITT principles were limited. Significant gaps were noted in methodological quality, particularly in stakeholder involvement and applicability. To enhance usability, guidelines should standardise recommendations for type, duration, intensity, and frequency, incorporating evidence grading system.

## Introduction

Cardio-respiratory diseases represent a significant global health burden, leading to some of the highest rates of morbidity and premature death worldwide.^1,2^ In the United Kingdom alone, nearly 7 million people live with cardio-respiratory conditions such as chronic obstructive pulmonary disease (COPD), asthma and heart failure.^3^ Strong evidence highlights that physical activity, in combination with dietary changes, smoking cessation, and sleep regulation, is central to cardio-respiratory disease management.^4–9^

Cardiac and pulmonary rehabilitation are evidence-based interventions for managing cardio-respiratory diseases, with exercise as a core component.^10–12^ Compared with general physical activity, rehabilitation programmes offer greater tailoring and patient-centred support. However, evidence for long-term improvements in physical activity is inconsistent, and access to and acceptability of these programmes are not universal.^10–12^

Physical activity encompasses any bodily movement that increases energy expenditure, while exercise is a planned and structured subset, typically defined using the frequency, intensity, time and type (FITT) framework. It is a cornerstone of lifestyle management, offering wide-ranging physical and mental health benefits across populations.^13–15^ However, individuals with cardio-respiratory diseases face particular challenges in achieving recommended physical activity levels. Symptoms such as breathlessness, fatigue, and exercise intolerance often limit their ability to meet general guidelines.^16^ This highlights the need for disease-specific guidance that considers both functional limitations and the changing nature of symptoms.^15–17^

Despite this need, there is a lack of recommendations tailored to disease-specific FITT principles, leaving healthcare professionals and patients uncertain about how to safely and effectively incorporate physical activity into disease management. Additionally, clinicians often hesitate to offer physical activity advice due to uncertainties about its safety in clinical care.^18^ Although numerous organisations have developed disease-specific evidence-based treatment guidelines, physical activity promotion is often missing. Furthermore, there remains an important knowledge gap because, where available, international physical activity guidelines for different cardio-respiratory diseases have not been synthesised.

Given the high prevalence and impact of cardio-respiratory diseases, chronic conditions such as COPD, asthma, and heart failure are among the most common, contributing to poor quality of life and reduced life expectancy.^1–3^ Despite their burden, there remains a lack of synthesised physical activity guidelines tailored to these conditions. Therefore, the objective of this study was to systematically review and consolidate physical activity guidelines for individuals with COPD, asthma, and heart failure (HF), with a specific focus on synthesising recommendations related to the FITT principle. Additionally, this study aimed to identify safety considerations and adaptation strategies for these individuals while highlighting gaps and limitations in current guidelines.

## Methods

We followed the PRISMA guidelines to structure our methods and reporting.^19^ This systematic review did not involve direct research with human or animal subjects and therefore was exempt from Institutional Review Board approval. This review was prospectively registered with the International Prospective Register of Systematic Reviews. The original PROSPERO registration (PROSPERO 2022, registration no. CRD42022309969) included respiratory, cardiovascular, and metabolic disease groups; due to the volume of data, the results are presented in separate publications. This manuscript focuses on cardio-respiratory diseases. This review addresses the following research questions: (a) what physical activity recommendations, including FITT are provided for individuals with COPD, asthma, or heart failure; and (b) what precautions and adaptations for physical activity are recommended for these conditions.

### Search strategy and study selection

Online databases OVID MEDLINE, EMBASE, CINAHL database were searched using a search strategy developed in consultation with a librarian specialising in Health Sciences. We searched for clinical guidelines from international and national organisations and from Google string searches. Additionally, we consulted experts in the fields of physical activity endocrinology to sense check our search strategy. Search strategies were developed for all databases individually. Variant combinations of MeSH terms and keywords was used with Boolean operators and truncation like ‘cardiovascular disease’, ‘physical activity’, ‘exercise’, ‘guideline’ ‘position statement’. To ensure that the review reflected the most recent evidence, a rapid updated search was conducted in MEDLINE (Ovid) after completion of the manuscript peer-review process and prior to publication. Newly identified records were screened using the same eligibility criteria applied in the original review. In addition, we checked for updated versions of the guidelines previously included in the review. Search strategies are presented in Supplementary Material 1.

Guidelines were included if they were international, national, or other expert consensus guidelines on physical activity and/or exercise; were produced by governmental or non-governmental organisations or unaffiliated expert groups; were published in English; and were developed for adults with COPD, asthma, or heart failure. Where available, the most recently updated versions of the guidelines were included. However, if a later version contained substantially different content or provided less information relevant to our focus on physical activity recommendations, any of earlier version was retained for analysis.

Guidelines were excluded if they were published before January 2000; were not sanctioned by a professional organisation or published in peer-reviewed journals; primarily focused on cardiac rehabilitation, pulmonary rehabilitation, clinical exercise interventions, supervised exercise therapy, or sport activities; focused on disease assessment or prevention rather than physical activity; were unrelated to physical activity recommendations; or were not specifically focused on COPD, asthma, or heart failure.

Documents were classified into six types. Guidelines are systematically developed documents providing recommendations to inform healthcare, public health, or policy decisions. Clinical practice guidelines focus on supporting clinical decision-making for patient diagnosis, treatment, or management. Statements outline expert consensus or principles on a specific topic, with or without formal evidence synthesis. Position papers present the official viewpoint of an organisation or professional body on an issue. Reports or consultations summarise evidence or stakeholder input to inform practice or policy without issuing formal recommendations. Strategies outline planned actions, priorities, and approaches to achieve specific health or policy objectives.

### Data extraction

Guidelines or recommendations identified from databases, as well as grey literature, were imported to reference management software. We extracted the following data: (a) FITT (Frequency, Intensity, Time, Type) physical activity recommendations. Two authors screened the titles and abstracts to identify the potential recommendations. Then, two authors (ET&HW) screened the full-text guidelines according to the eligibility criteria. The reasons for excluding guidelines or recommendations were documented. Two authors consulted with a third reviewing author (RE) to resolve any disagreements during the guideline inclusion process. Recommendations were categorised as FITT and physical activity. Adaptations and precautions were also being recorded. Guideline name, country, issuing authority, and date were also noted.

### Risk of bias (quality) assessment

The methodological quality of the clinical practice guidelines was evaluated by the AGREE II instrument^20^ (https://www.agreetrust.org/agree-ii/). We evaluated quality, including 23 items in 6 areas and one overall assessment items within AGREE II. The minimum score for each item was 1, and the maximum score was 7. We calculated the final score of each field according to the formula as follows: each field score = (actual score - minimum possible score)/(maximum possible score - minimum possible score) × 100. The following 6 domains were assessed by two authors: scope and purpose; stakeholder involvement; rigour of development; clarity of presentation; applicability; editorial independence.

Thresholds for quality classification (high, moderate, low) were established through consensus, as follows:

- *High quality*: All domains achieving a score of at least 60%.
- *Moderate quality*: At least 5 domains scoring over 50%, with no domains falling below 30%.
- *Low quality*: Guidelines failing to meet the aforementioned criteria.

## Results

**Figure 1.**
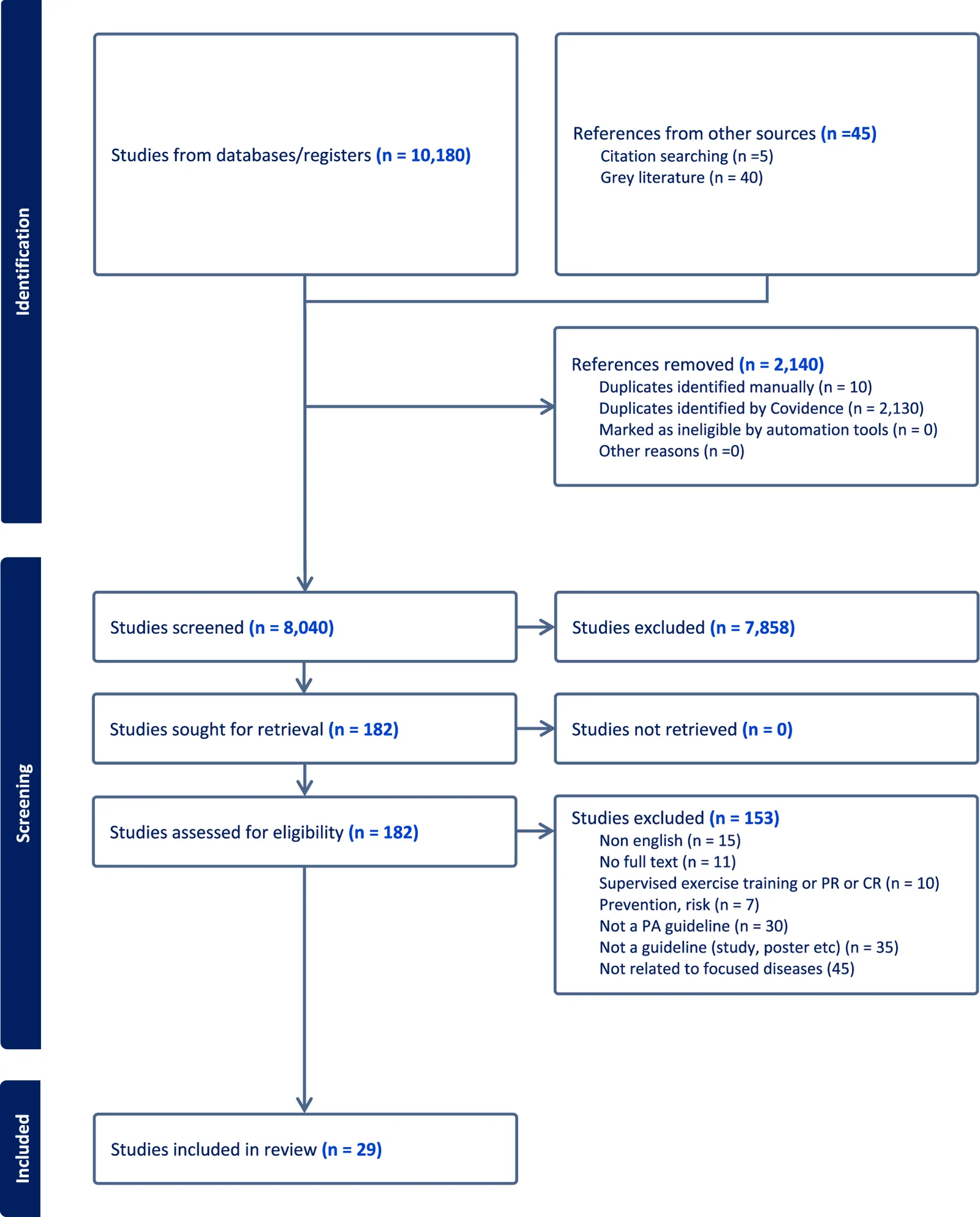
PRISMA flow diagram.

The initial search of four electronic databases identified 10,180 citations. Several citations were excluded for various reasons, such as not being official guidelines or consensus documents, not being related to physical activity, lacking full-text access, or focusing on clinical exercise rehabilitation recommendations. In total, 29 guidelines met the inclusion criteria for our study.^21–49^ Figure 1 presents the PRISMA flow diagram, illustrating the review process. A comprehensive systematic search was conducted in June 2025, followed by a rapid updated search in March 2026.

A total of 29 clinical guidelines were included: 11 focused on COPD, 11 on asthma, and 7 on heart failure.^21–49^ The characteristics and development processes of these guidelines are summarised in Table 1.

**Table 1.** Characteristics of guidelines.

	Organization/Institution	Year	Origin	Type	Own recs	Grade recs	Reference
COPD							
1	Indian CPG	2013	India	CPG	yes	yes	^21^
2	Italian CPG	2014	Italy	CPG	yes	no	^22^
3	ERS	2014	Europe	Statement	no	no	^23^
4	V/DoD	2014	US	CPG	yes	yes	^24^
5	BCG	2017	Canada	Guideline	yes	no	^25^
6	NICE	2018	UK	Guideline	yes	no	^26^
7	ESSA	2021	Australia	Statement	no	no	^27^
8	GesEPOC	2022	Spain	Guideline	yes	no	^28^
9	JRS	2022	Japan	CPG	yes	Yes	^29^
10	COPD-X	2025	Australia/NZ	CPG	yes	yes	^30^
11	GOLD	2026	Global	Strategy Report	yes	no	^31^
ASTHMA							
1	NAEPP	2007	USA	Guideline	Yes	No	^32^
2	AAESS	2011	Australia	Statement	Yes	No	^33^
3	JGAA	2014	Japan	Guideline	No	No	^34^
4	IACBP	2017	Global	CPG	Yes	Yes	^35^
5	SIGN	2019	UK	CPG	Yes	Yes	^36^
6	NZ-AARF	2020	New Zealand	CPG	Yes	No	^37^
7	AAAAI	2022	US	Report	Yes	No	^38^
8	SIA	2024	Saudi Arabia	CPG	No	Yes	^39^
9	BCG	2024	Canada	Guideline	Yes	No	^40^
10	GINA	2025	Global	Strategy Report	Yes	Yes	^41^
11	EAACI	2025	European	Statement	Yes	Yes	^42^
HF							
1	AHA	2015	USA	Call to Action	No	No	^43^
2	CAG	2016	Canada	Stakeholder consultation	No	No	^44^
3	CCS	2017	Canada	CPG	Yes	Yes	^45^
4	FSC	2019	France	Position Paper	Yes	No	^46^
5	ESC1	2020	Europe	GPG	Yes	Yes	^47^
6	ESC2	2021	Europe	GPG	Yes	Yes	^48^
7	AHA/ACC/HFSA	2022	USA	CPG	Yes	Yes	^49^

*Abbreviations*: Indian (Indian COPD guideline by Indian Chest Society and National College of Chest Physicians); Italian (Italian COPD guidelines by Interdisciplinary Association for Research in Lung Disease and Italian Association of Hospital Pulmonologists, Italian Society of Respiratory Medicine, Italian Society of General Medicine); ERS (European Respiratory Society); VA/DoD (Veteran Affairs/Department of Defence guideline); COPD-X (Guidelines for the Management of. Chronic Obstructive Pulmonary Disease); GOLD (Global Strategy for the Diagnosis, Management, and Prevention of Chronic Obstructive Lung Disease); BCG (British Columbia Guidelines); NICE (National Institute Health and Care Excellence); JRS (Japanese Respiratory Society); ESSA (Exercise & Sports Science Australia); GesEPOC (Spanish COPD Guidelines) NAEPP (National Asthma Education and Prevention Program of National Heart, Lung, and Blood Institute); AAESS (Australian Association for Exercise and Sports Science); JGAA (Japanese Guideline for Adult Asthma); IACBP (International Affairs & Clinical Best Practice Guidelines); SIGN (Scottish Intercollegiate Guidelines Network); NZ-AARF (Asthma and Respiratory Foundation New Zealand); AAAAI (American Academy of Allergy, Asthma, and Immunology); SIA (The Saudi Initiative for Asthma); BCG (British Columbia Guidelines); GINA (Global Initiative for Asthma); EAACI (European Academy of Allergy Clinical Immunology) AHA (American Heart Association); CAG (Canadian Journal on Gerontology); CCS (Canadian Cardiovascular Society); FSC (French Society of Cardiology); ESC (European Society of Cardiology); AHA/ACC/HFSA (American College of Cardiology/American Heart Association/ Heart Failure Society America). Own Recs; Recommendations formulated by the guideline development group based on their own evidence synthesis or expert consensus. Grade Recs; Recommendations with formally graded strength and/or certainty of evidence.

Guidelines were developed by a wide range of national and international organisations. COPD guidelines originated from Europe, North America, Asia, and Australia, with contributions from international bodies as well as country-specific groups. Asthma guidelines showed a similar pattern, with a mix of global organisations and national guideline developers across multiple regions. Heart failure guidelines were primarily produced by European and North American organisations. Earlier versions of the V/Dod, BCG, GesEPOC, JGAA, and SIGN guidelines were included, as the more recent versions either did not include physical activity recommendations or provided limited information on physical activity adaptations.

### Physical activity recommendations

Fourteen of the 29 guidelines were classified as clinical practice guidelines. Most reported conducting systematic literature searches, although approaches to evidence appraisal and synthesis varied considerably between guideline groups.

Across COPD, asthma, and heart failure, all guidelines encouraged regular physical activity as part of long-term disease management.

Of the 29 included guidelines, 11 provided aerobic physical activity recommendations aligned with FITT principles, including 4 for COPD, 3 for asthma, and 4 for heart failure.^27,28,30,31,33,38,42,43,45,47,48^ These guidelines generally recommended approximately 150 min of moderate-to-vigorous physical activity per week, typically distributed across 3 to 5 days. For COPD, only four guidelines offered explicit FITT-based recommendations.^27,28,30,31^ Most other guidelines encouraged regular or daily activity without defining specific parameters, framing physical activity within self-management and patient education during periods of stable disease. Asthma guidelines generally promoted physical activity but provided limited FITT-based guidance until recently; only 3 of the 11 guidelines included FITT-based recommendations focused on aerobic activity. Notably, the most recent European asthma guideline provided the most comprehensive FITT-based guidance for this population.^42^ All heart failure guidelines recommended engagement in physical activity and exercise, with more than half supporting the 150-minute weekly target.^43,45,47,48^

There was limited representation across the 29 guidelines for strength, endurance, or flexibility exercises, with only 5 providing recommendations for strength and endurance,^30,31,42,45,48^ while only 1 recent guideline included guidance for flexibility-type of exercise. Notably, detailed FITT guidance for strength, endurance, and flexibility was provided in the most recent European asthma guideline.^45^ Additionally, one COPD guideline recommended a combination of aerobic, strength, and interval-based training.^31^

Across all conditions, guidelines emphasised tailoring physical activity to individual symptoms, disease stability, and daily functional needs. Precautions and adaptations were not standardised across guidelines, and the specific recommendations varied by condition. Guidance on pre-exercise testing or formal medical clearance was limited.^32,41,42,47^

In COPD, adaptations focused on managing exacerbations, optimising medication use, promoting pulmonary rehabilitation, and supporting self-management and community-based programmes.^26–31^ Environmental considerations, including air quality, the use of short-acting beta2-agonists to improve exercise tolerance, and follow-up assessments were emphasised, alongside strategies for older adults, motivational and digital support, and monitoring exercise intensity.^25,31^

For asthma, guidelines primarily addressed adaptations to prevent exercise-induced symptoms and manage environmental triggers, including air quality, pollen exposure, temperature, and humidity.^32–34^^,36,37,39,41,42^ Recommendations included consulting healthcare professionals when symptom control was a concern, use of reliever bronchodilators, warm-up routines, motivational support, tailoring programmes for older adults, and monitoring intensity.^32,33,36,39,42^

In heart failure, recommendations emphasised safe engagement in physical activity, clinical reassessment when increasing intensity, integration of physical activity into daily routines, individualised exercise prescriptions, and structured exercise-based cardiac rehabilitation to improve functional capacity.^44,46–50^ Additional strategies included motivational and psychological support, adaptations for comorbidities and symptoms, and monitoring of exercise intensity.^44,47^

Tables 2 to 4 summarise condition-specific recommendations, with further detail provided in Supplementary Material 2.

**Table 2.** Physical activity recommendations: COPD.

	Indian	Italian	ERS	V/Dod	BCG	NICE	ESSA	GesEPOC	JRS	COPD-X	GOLD
**General PA**											
Regular PA encouraged	✓	✓	✓	✓	✓	✓	✓	✓	✓	✓	✓
PA for stable COPD						✓					✓
PA in self-management/education			✓	✓							✓
**Aerobic FITT**											
≥150 min/week aerobic PA							✓	✓		✓	
Spread over 3–5 days/week							✓			✓	
Moderate–vigorous intensity (60–80% HR, Borg 4–6)										✓	✓
Start at 80% of 6MWT							✓				
**Strength/Endurance**											
At least 2 days a week										✓	
Combine aerobic, strength & intervals											✓
**Precautions and adaptations**											
Exacerbation considerations						✓	✓				
Daily living/symptom/comorbidity adaptations				✓				✓			✓
Bronchodilators for tolerance								✓			✓
PR to improve dyspnoea/exercise				✓		✓			✓	✓	✓
Community-based PA											✓
Air quality advice					✓						
Follow-up PA assessment											✓

*Abbreviations*: Indian (Indian COPD guideline by Indian Chest Society and National College of Chest Physicians); Italian (Italian COPD guidelines by Interdisciplinary Association for Research in Lung Disease and Italian Association of Hospital Pulmonologists, Italian Society of Respiratory Medicine, Italian Society of General Medicine); ERS (European Respiratory Society); VA/DoD (Veteran Affairs/Department of Defence guideline); COPD-X (Guidelines for the Management of. Chronic Obstructive Pulmonary Disease); GOLD (Global Strategy for the Diagnosis, Management, and Prevention of Chronic Obstructive Lung Disease); BCG (British Columbia Guidelines); NICE (National Institute Health and Care Excellence); JRS (Japanese Respiratory Society); ESSA (Exercise & Sports Science Australia); GesEPOC (Spanish COPD Guidelines); PA (Physical Activity).

**Table 3.** Physical activity recommendations: Asthma.

Section	NAEPP	AAESS	JGAA	IACBP	SIGN	NZ-AARF	AAAAI	SIA	BCG	GINA	EAACI
General PA encouraged	✓	✓	✓	✓	✓	✓	✓	✓	✓	✓	✓
Aerobic FITT											
Gradually increase to ≥30 min/day of preferred activity, most days.							✓				
3–5 days/week, moderate–vigorous intensity, 30–40 min (e.g., walking, cycling)											✓
20–60 min aerobic exercise		✓									
Strength Endurance/Flexibility											
2–3 days/week: Strength (60–70% 1RM), Endurance (<50% 1RM), Flexibility (yoga, Pilates).											✓
Precautions and adaptations							✓			✓	✓
Warm up before exercise	✓						✓			✓	✓
Use SABA/ICS pre-exercise	✓	✓	✓		✓	✓	✓	✓	✓	✓	✓
Avoid cold, allergens, pollution							✓	✓		✓	✓
Medical clearance before PA	✓						✓				✓
Use digital/motivational support							✓				✓
Address beliefs/barriers							✓				
Tailor PA for older adults							✓				✓
Use Borg scale/Talk Test											✓
Monitor environment											✓
Choose indoor/HEPA options											✓
Wear mask in cold/pollution											✓
Consider altitude therapy (severe cases)											✓

*Abbreviations*: NAEPP (National Asthma Education and Prevention Program of National Heart, Lung, and Blood Institute); AAESS (Australian Association for Exercise and Sports Science); JGAA (Japanese Guideline for Adult Asthma); IACBP (International Affairs & Clinical Best Practice Guidelines); SIGN (Scottish Intercollegiate Guidelines Network); NZ-AARF (Asthma and Respiratory Foundation New Zealand); AAAAI (American Academy of Allergy, Asthma, and Immunology); SIA (The Saudi Initiative for Asthma); BCG (British Columbia Guidelines); GINA (Global Initiative for Asthma); EAACI (European Academy of Allergy Clinical Immunology); SABA (Short-Acting Beta-2 Agonist); ICS (Inhaled Corticosteroids); HEPA (High Efficiency Particulate Air Filter); PA (Physical Activity).

**Table 4.** Physical activity recommendations: Heart failure.

	AHA	CAG	CCS	FSC	ESC1	ESC2	AHA/ACC/HFSA
General PA							
Regular PA for stable COPD; reduces hospitalisation, improves QoL	✓	✓	✓	✓	✓	✓	✓
Aerobic FITT							
≥150 min/week	✓		✓		✓	✓	
Moderate intensity	✓		✓		✓	✓	
Moderate-to-vigorous intensity	✓					✓	
Spread over 3–5 days/week	✓		✓		✓	✓	
Strength and Endurance							
30 min resistance training			✓				
10–20 reps with 5–10 lb weights			✓			✓	
2–3 days/week			✓			✓	
Borg Scale <15			✓				
Precautions and adaptations							
Adaptation for symptoms, comorbidities, daily needs		✓			✓	✓	
Motivational support					✓		
Repeat exercise test at higher intensity					✓		
Monitor ventricular rate before/during exercise			✓				
Avoid Sports Especially with anticoagulants					✓		
Cardiac rehab recommended for stable patients						✓	✓
HIIT may suit low-risk patients returning to sport					✓		

*Abbreviations*: AHA (American Heart Association); CAG (Canadian Journal on Gerontology); CCS (Canadian Cardiovascular Society); FSC (French Society of Cardiology); ESC (European Society of Cardiology); AHA/ACC/HFSA (American College of Cardiology/American Heart Association/ Heart Failure Society America); PA (Physical Activity).

### Methodological quality of recommendations

Among the 14 guidelines, seven were rated as high quality across multiple domains, six as moderate quality, and one as low quality. The highest-quality guidelines identified for each condition were the 2024 Australian and New Zealand guideline for COPD, the 2019 United Kingdom guideline for asthma, and the 2022 American guideline for heart failure.^30,36,49^ These guidelines consistently achieved the highest scores across AGREE II domains, indicating strong methodological rigour and reliable recommendations for clinical practice. In contrast, the lowest-scoring domains across most guidelines were stakeholder involvement and applicability. Table 5 presents the AGREE-II domain scores for each of the 14 clinical practice guidelines. Overall, the mean scores of all domains exceeded 60%. Kappa scores greater than 0.7 indicate a moderate-to-strong level of agreement between the two assessors.

**Table 5.** Quality assessment of guidelines.

Organisation		Domain^a^ scores (%)						Mean scores	Agreement, kappa coefficient	Quality
		D1	D2	D3	D4	D5	D6			
Heart failure	CCS	100	83	94	94	64	100	89	0.96	High
	ESC2	100	69	100	100	45	100	86	0.87	Moderate
	ESC1	100	69	100	100	45	100	86	1.00	Moderate
	AHA/ACC/HFSA	100	83	92	100	88	100	94	0.93	High
COPD	Indian CPG	86	67	81	83	17	75	68	0.78	Moderate
	Italian CPG	67	33	29	17	28	75	41	0.78	Low
	V/DoD	100	100	94	83	83	100	93	0.96	High
	COPD-X	100	67	100	100	100	100	95	1.00	High
	GOLD	96	58	75	76	36	57	66	0.96	Moderate
Asthma	IACBP	100	100	94	100	100	100	99	0.96	High
	SIGN	100	100	100	100	100	100	100	1.00	High
	NZ-AARF	92	83	65	69	33	100	74	0.70	Moderate
	SIA	100	83	94	83	88	100	91	0.91	High
	GINA	95	58	75	90	45	55	70	0.91	Moderate

a Domains: D1 (scope and purpose), D2 (stakeholder involvement), D3 (rigour of development) D4 (clarity of presentation), D5 (applicability), D6 (editorial independence), D7 (general quality).

*Abbreviations*: Indian (Indian COPD guideline by Indian Chest Society and National College of Chest Physicians); Italian (Italian COPD guidelines by Interdisciplinary Association for Research in Lung Disease and Italian Association of Hospital Pulmonologists, Italian Society of Respiratory Medicine, Italian Society of General Medicine); COPD-X (Guidelines for the Management of Chronic Obstructive Pulmonary Disease); GOLD (Global Strategy for the Diagnosis, Management, and Prevention of Chronic Obstructive Lung Disease); IACBP (International Affairs & Clinical Best Practice Guidelines); SIGN (Scottish Intercollegiate Guidelines Network); NZ-AARF (Asthma and Respiratory Foundation New Zealand); AAAAI (American Academy of Allergy, Asthma, and Immunology); SIA (The Saudi Initiative for Asthma); GINA (Global Initiative for Asthma); CCS (Canadian Cardiovascular Society); ESC (European Society of Cardiology); AHA/ACC/HFSA (American College of Cardiology/American Heart Association/ Heart Failure Society America).

### Updated search

The updated search identified a total of 306 new records, of which nine guidelines were selected for full-text screening. After full-text assessment, none met the eligibility criteria for inclusion in the review. We also checked for updated versions of the guidelines previously included in the review. The most recent versions identified were COPD-X 2025 and GOLD 2026. These updated guidelines have been incorporated into our study.

## Discussion

We conducted a novel, large-scale synthesis of physical activity recommendations in cardio-respiratory disease guidelines, focusing on COPD, asthma, and heart failure. While evidence-based guidelines generally promote an active lifestyle for managing these conditions, specific guidance using the FITT principles was often lacking. Recommendations were frequently vague and generic, resembling those for the general population, with limited condition-specific precautions or adaptations. Only four COPD and three asthma guidelines provided detailed aerobic FITT recommendations, with asthma guidelines tending to emphasise disease complications rather than general physical activity advice. In contrast, more than half of heart failure guidelines included aerobic FITT guidance. Strength, endurance, and flexibility recommendations were poorly represented, with only five guidelines providing any guidance. These findings highlight the need for clearer, tailored physical activity recommendations for both healthcare professionals and patients.

Although the health benefits of physical activity for cardio-respiratory diseases are well established, our review found that FITT-based recommendations are limited, with only 11 of 29 guidelines providing specific guidance on frequency, intensity, time, or type. This gap may reflect limited evidence on how variations in intensity, duration, and type specifically affect individuals with these conditions and a lack of consensus on relevant outcome measures in real-world settings.^50–52^ However, while developing precise FITT prescriptions for this population is challenging, the inclusion of minimum or pragmatic FITT guidance may improve the implement ability of physical activity advice. Providing disease-specific precautions and adaptations, as reported in this review, alongside FITT-based recommendations may enhance the clarity, safety, and practical usefulness of physical activity guidance for both healthcare professionals and patients.

Our review found that strength or resistance or recommendations are underrepresented in physical activity guidelines for cardio-respiratory patients.^30,31,42,45,48^ This may be because resistance exercise was historically under-prescribed due to concerns about increased heart workload and blood pressure,^53,54^ although these concerns have since been disproven.^55,56^ Evidence now indicates that combining resistance with aerobic training in clinically stable patients is safe and provides benefits.^55,56^ Notably, a very recent statement included in our review from the provides the most detailed guidance to date on resistance-based physical activity, which may encourage future guidelines to address this component more comprehensively.^42^

Overall, the guidelines demonstrate that while physical activity is universally encouraged across cardio-respiratory conditions, the way adaptations and precautions are framed differs substantially by disease context. Respiratory conditions place greater emphasis on symptom self-management, use of bronchodilator therapy, environmental exposures, whereas heart failure guidance prioritises safety, structured progression.^21–49^ Although standardisation is challenging due to differences in disease severity, symptom burden, and individual characteristics, the focus areas for physical activity precautions and adaptations within guidelines should at least be standardised. For example, core domains such as safety, symptom-guided exercise, bronchodilator use, and environmental factors including air pollution and weather conditions should be consistently addressed.

Exercise-based cardiopulmonary rehabilitation provides well-established benefits for adults with COPD, asthma, and heart failure and may support improvements in physical activity levels.^57–59^ However, our review identified limited guidance on rehabilitation within general disease management guidelines.^24,26,29–31^^,48,49^ While more detailed recommendations may be available in dedicated rehabilitation guidelines, general guidelines should emphasise the role of rehabilitation as a foundation for promoting physical activity and provide clearer guidance on transitioning from rehabilitation to sustained physical activity engagement.

Regarding the quality of the guidelines, limitations were noted in areas such as stakeholder involvement and applicability. Extracting comprehensive FITT recommendations for physical activity was challenging due to the heterogeneity in reporting. Directly integrating FITT a widely accepted framework for prescribing physical activity into the guidelines could greatly enhance clarity and practicality, ultimately improving their quality. Additionally, there were instances where it was unclear whether specific instructions were intended solely for the general adult population or if they also included recommendations relevant to the target audience of this article. Therefore, involving users in the development stage, alongside editorial review, is essential.

This study has several key strengths and limitations that should be considered when interpreting the findings. In addition to searching electronic databases, we also explored clinical guideline databases, reference lists of systematic reviews, and websites of authoritative bodies in the field and consult to experts which is for dealing with challenging of the guideline search from official sites can be seen as strength. Another important strength is the use of the AGREE II instrument, which enabled a rigorous and transparent assessment of guideline quality. Building on this, future work could apply AGREE-REX to assess the implementability of individual guideline recommendations, which would further enhance their translation into clinical practice.^60^ However, several limitations should be acknowledged. First, we excluded guidelines that were not available in English or did not have an accessible English version, which may have led to the omission of relevant guidance from non-English-speaking contexts. Second, not all cardio-respiratory diseases were included in this review.

In conclusion, while the guidelines consistently recommended aerobic physical activity, they lacked sufficient disease specificity and clear application of the FITT principles. Important limitations were identified in the methodological quality of the guidelines, particularly in relation to stakeholder involvement and applicability domains. To enhance their practical use for healthcare professionals and patients, guidelines should standardise recommendations regarding the type, duration, intensity, and frequency of physical activity, with clear evidence grading system.

**Clinical messages**

- Cardio-respiratory guidelines promote physical activity but often lack clear recommendations on the FITT of physical activity.
- Standardised recommendations on the FITT of physical activity are needed to improve clarity, clinical implementation, and patient outcomes.
- Physical activity recommendations should consistently integrate key adaptations and precautions including safety, symptom-guided exercise, bronchodilator use, and environmental factors while remaining responsive to disease-specific needs and individual characteristics.
- Rehabilitation should be emphasised as the foundation for physical activity promotion, with clearer guidance on transitioning from rehabilitation to sustained long-term engagement.

## Supplemental Material

sj-docx-1-cre-10.1177_02692155261441158 - Supplemental material for A systematic review of physical activity guidelines for adults with cardio-respiratory diseases: Stepping towards evidence-based recommendations Supplemental material, sj-docx-1-cre-10.1177_02692155261441158 for A systematic review of physical activity guidelines for adults with cardio-respiratory diseases: Stepping towards evidence-based recommendations by Eda Tonga, Thomas Yates, Hannah Worboys, Sally J Singh, Pip Divall, G Andre Ng and Rachael A Evans in Clinical Rehabilitation

sj-docx-2-cre-10.1177_02692155261441158 - Supplemental material for A systematic review of physical activity guidelines for adults with cardio-respiratory diseases: Stepping towards evidence-based recommendations Supplemental material, sj-docx-2-cre-10.1177_02692155261441158 for A systematic review of physical activity guidelines for adults with cardio-respiratory diseases: Stepping towards evidence-based recommendations by Eda Tonga, Thomas Yates, Hannah Worboys, Sally J Singh, Pip Divall, G Andre Ng and Rachael A Evans in Clinical Rehabilitation
