# Supplementary material for: A systematic review of physical activity guidelines for adults with cardio-respiratory diseases: Stepping towards evidence-based recommendations
Source: Clin Rehabil. 2026 Apr 24;40(9):1143–59. doi: 10.1177/02692155261441158 (PMC13525178; doi:10.1177/02692155261441158)
Supplement: Supplementary file 1 [file sj-docx-1-cre-10.1177_02692155261441158.docx]

**Supplementary material 1:**

A) **Systematic Review Strategies**

**Original search**

| **Database** | **Provider** | **Total No. of results** | **(2025 update)** |
| --- | --- | --- | --- |
| MEDLINE | Ovid | 5399 | (982) |
| Embase | Ovid | 9711 | (2440) |
| CINAHL | EBSCOHost | 7150 | (1127) |

Limits applied: 2000-date

Methodological/subject filters applied:
CADTH filter for guidelines:

[Search Results - Search Filters Database - Canada's Drug Agency](https://searchfilters.cda-amc.ca/list?q=&p=1&ps=20&topic_facet=guidelines%20000000%7CGuidelines) (accessed 17/6/25)

Embase <1974 to 2025 June 27>

1	fitness/	50190

2	exp exercise/	525699

3	kinesiotherapy/	44756

4	cardiorespiratory fitness/	12147

5	sedentary lifestyle/	26960

6	(physical adj2 activit*).ti,ab,kw,kf.	259069

7	exercis*.ti,ab,kw,kf.	580692

8	running/ or jogging/ or (running or jogging).ti,ab,kw,kf.	117341

9	Swimming/ or Swimming.ti,ab,kw,kf.	53551

10	(walking or stair climbing).ti,ab,kw,kf.	150877

11	walking/ or stair climbing/	104757

12	(sedentary adj2 behavio?r*).ti,ab,kw,kf.	16457

13	(sedentary adj2 lifestyle*).ti,ab,kw,kf.	11082

14	(physical adj2 inactiv*).ti,ab,kw,kf.	17789

15	or/1-14	1189263

16	cardiovascular disease/	423584

17	(coronary heart disease or CHD or coronary artery disease or CAD).ti,ab,kw,kf.	339564

18	coronary artery disease/	264565

19	(chronic heart failure or CHF).ti,ab,kw,kf.	58130

20	heart failure/	372429

21	exp Asthma/ or asthma.ti,ab,kw,kf.	375097

22	(chronic obstructive pulmonary disease or COPD).ti,ab,kw,kf.	165528

23	(chronic and obstructive and (airway or respiratory or lung) and disease).ti,ab,kw,kf.	70490

24	chronic obstructive lung disease/	210108

25	(Interstitial lung disease or ILD).ti,ab,kw,kf.	38515

26	exp interstitial lung disease/	156860

27	(cardiovascular disease* or CVD).ti,ab,kw,kf.	421922

28	or/16-27	1976354

29	exp clinical pathway/	11142

30	exp clinical protocol/	139112

31	clinical protocols/	124102

32	exp consensus/	113514

33	exp consensus development conference/	31164

34	exp consensus development conferences as topic/	31164

35	critical pathways/	11142

36	guidelines as topic/	610514

37	exp practice guideline/	828346

38	practice guidelines as topic/	610514

39	health planning guidelines/	115452

40	Clinical Decision Rules/	1265

41	(guideline or practice guideline or consensus development conference or consensus development conference, NIH).pt.	0

42	(position statement* or policy statement* or practice parameter* or best practice*).ti,ab,kf,kw.	81429

43	(standards or guideline or guidelines).ti,kf,kw.	206763

44	((practice or treatment* or clinical) adj guideline*).ab.	99329

45	(CPG or CPGs).ti.	8242

46	consensus*.ti,kf,kw.	51806

47	((critical or clinical or practice) adj2 (path or paths or pathway or pathways or protocol*)).ti,ab,kf,kw.	50860

48	(guideline* or standards or consensus* or recommendat*).au.	29

49	(guideline* or standards or consensus* or recommendat*).co.	2435

50	chronic disease/	223730

51	("chronic disease*" or "long term condition*" or "long term disease*").mp.	318768

52	exp *adolescence/ or exp *adolescent/ or exp *child/ or exp *childhood disease/ or exp *infant disease/ or (adolescen* or babies or baby or boy? or boyfriend or boyhood or girlfriend or girlhood or child or child* or child*3 or children* or girl? or infan* or juvenil* or juvenile* or kid? or minors or minors* or neonat* or neo-nat* or newborn* or new-born* or paediatric* or peadiatric* or pediatric* or perinat* or preschool* or puber* or pubescen* or school* or teen* or toddler? or underage? or under-age? or youth*).ti,kw.	3614792

53	52 not adult*.mp.	2616758

54	"clinicaltrials.gov".pb.	533511

55	or/29-49	1254301

56	28 or 50 or 51	2244506

57	15 and 55 and 56	11898

58	57 not 54	10609

59	58 not 53	10133

60	limit 59 to yr="2000 -Current"	9711

61	limit 59 to dc=20220208-20250617	2440

Ovid MEDLINE(R) ALL <1946 to June 16, 2025>

1	Physical Fitness/	30907

2	exp Exercise/	273183

3	exp Exercise Therapy/	71273

4	Cardiorespiratory Fitness/	3981

5	Sedentary Behavior/	15532

6	(physical adj2 activit*).ti,ab,kw,kf.	182231

7	exercis*.ti,ab,kw,kf.	409184

8	exp Running/ or running.ti,ab,kw,kf. or jogging.ti,ab,kw,kf.	90016

9	Swimming/ or Swimming.ti,ab,kw,kf.	45879

10	walking/ or stair climbing/	45509

11	(walking or stair climbing).ti,ab,kw,kf.	103415

12	(sedentary adj2 behavio?r*).ti,ab,kw,kf.	13329

13	(sedentary adj2 lifestyle*).ti,ab,kw,kf.	6939

14	(physical adj2 inactiv*).ti,ab,kw,kf.	12607

15	or/1-14	796980

16	exp Cardiovascular Diseases/	2874846

17	(coronary heart disease or CHD or coronary artery disease or CAD).ti,ab,kw,kf.	209639

18	Coronary Disease/ or Coronary Artery Disease/	213049

19	(chronic heart failure or CHF).ti,ab,kw,kf.	31258

20	exp Heart Failure/	160356

21	exp Asthma/ or asthma.ti,ab,kw,kf.	212484

22	(chronic obstructive pulmonary disease or COPD).ti,ab,kw,kf.	90332

23	(chronic and obstructive and (airway or respiratory or lung) and disease).ti,ab,kw,kf.	43928

24	Pulmonary Disease, Chronic Obstructive/	55757

25	(Interstitial lung disease or ILD).ti,ab,kw,kf.	19350

26	exp Lung Diseases, Interstitial/	90571

27	(cardiovascular disease* or CVD).ti,ab,kw,kf.	283500

28	or/16-27	3417442

29	exp clinical pathway/	8345

30	exp clinical protocol/	201561

31	clinical protocols/	30508

32	exp consensus/	25444

33	exp consensus development conference/	13199

34	exp consensus development conferences as topic/	3021

35	critical pathways/	8345

36	guidelines as topic/	42651

37	exp practice guideline/	34371

38	practice guidelines as topic/	132774

39	health planning guidelines/	4169

40	Clinical Decision Rules/	1028

41	(guideline or practice guideline or consensus development conference or consensus development conference, NIH).pt.	51759

42	(position statement* or policy statement* or practice parameter* or best practice*).ti,ab,kf,kw.	55809

43	(standards or guideline or guidelines).ti,kf,kw.	152964

44	((practice or treatment* or clinical) adj guideline*).ab.	62850

45	(CPG or CPGs).ti.	6801

46	consensus*.ti,kf,kw.	41596

47	consensus*.ab. /freq=2	41358

48	((critical or clinical or practice) adj2 (path or paths or pathway or pathways or protocol*)).ti,ab,kf,kw.	32121

49	recommendat*.ti,kf,kw. or guideline recommendation*.ab.	68030

50	(care adj2 (standard or path or paths or pathway or pathways or map or maps or plan or plans)).ti,ab,kf,kw.	102305

51	(algorithm* adj2 (screening or examination or test or tested or testing or assessment* or diagnosis or diagnoses or diagnosed or diagnosing)).ti,ab,kf,kw.	12072

52	(algorithm* adj2 (pharmacotherap* or chemotherap* or chemotreatment* or therap* or treatment* or intervention*)).ti,ab,kf,kw.	14947

53	(guideline* or standards or consensus* or recommendat*).au.	11

54	(guideline* or standards or consensus* or recommendat*).ca.	3608

55	or/29-54	839802

56	Chronic Disease/	292239

57	("chronic disease*" or "long term condition*" or "long term disease*").mp. [mp=title, book title, abstract, original title, name of substance word, subject heading word, floating sub-heading word, keyword heading word, organism supplementary concept word, protocol supplementary concept word, rare disease supplementary concept word, unique identifier, synonyms, population supplementary concept word, anatomy supplementary concept word]	379529

58	56 or 57	379529

59	28 or 58	3712761

60	15 and 55 and 59	6090

61	(child* or stepchild* or step-child* or kid or kids or girl or girls or boy or boys or teen* or youth* or youngster* or adolescent* or adolescence or preschool* or pre-school* or kindergarten* or school* or juvenile* or minors or p?ediatric* or PICU).ti,ab. or exp child/	3510617

62	60 not 61	5399

63	("20220208" or "20220209" or 2022021* or 2022022* or 202203* or 202204* or 202205* or 202206* or 202207* or 202208* or 202209* or 202210* or 202211* or 202212* or 2023* or 2024* or 2025* or 2026*).dt,ez,ed.	5720893

64	62 and 63	982

CINAHL via EBSCOHostTop of Form

|  | Tue, June 17, 2025 02:11:51 pm |
| --- | --- |

| **#** | **Query** | **Limiters/Expanders** | **Results** |
| --- | --- | --- | --- |
| S1 | (MH "Physical Fitness+") | Expanders - Apply equivalent subjects Search modes - Proximity | 22,754 |
| S2 | (MH "Exercise+") | Expanders - Apply equivalent subjects Search modes - Proximity | 136,788 |
| S3 | (MH "Therapeutic Exercise") | Expanders - Apply equivalent subjects Search modes - Proximity | 30,629 |
| S4 | (MH "Cardiorespiratory Fitness") | Expanders - Apply equivalent subjects Search modes - Proximity | 2,151 |
| S5 | physical n2 activit* | Expanders - Apply equivalent subjects Search modes - Proximity | 108,271 |
| S6 | exercis* | Expanders - Apply equivalent subjects Search modes - Proximity | 226,108 |
| S7 | (MH "Running+") OR "Running" | Expanders - Apply equivalent subjects Search modes - Proximity | 26,501 |
| S8 | (MH "Jogging") OR "jogging" | Expanders - Apply equivalent subjects Search modes - Proximity | 820 |
| S9 | (MH "Swimming") OR "swimming" | Expanders - Apply equivalent subjects Search modes - Proximity | 7,231 |
| S10 | (MH "Stair Climbing") OR (MH "Walking") OR "walking OR stair climbing" | Expanders - Apply equivalent subjects Search modes - Proximity | 27,367 |
| S11 | sedentary n2 behavio?r* | Expanders - Apply equivalent subjects Search modes - Proximity | 2,112 |
| S12 | sedentary n2 lifestyle* | Expanders - Apply equivalent subjects Search modes - Proximity | 2,309 |
| S13 | physical n2 inactiv* | Expanders - Apply equivalent subjects Search modes - Proximity | 5,047 |
| S14 | S1 OR S2 OR S3 OR S4 OR S5 OR S6 OR S7 OR S8 OR S9 OR S10 OR S11 OR S12 OR S13 | Expanders - Apply equivalent subjects Search modes - Proximity | 355,173 |
| S15 | (MH "Cardiovascular Diseases+") | Expanders - Apply equivalent subjects Search modes - Proximity | 690,050 |
| S16 | coronary heart disease or CHD or coronary artery disease or CAD | Expanders - Apply equivalent subjects Search modes - Proximity | 80,369 |
| S17 | (MH "Coronary Disease") | Expanders - Apply equivalent subjects Search modes - Proximity | 24,960 |
| S18 | (MH "Coronary Arteriosclerosis") | Expanders - Apply equivalent subjects Search modes - Proximity | 23,120 |
| S19 | chronic heart failure or CHF | Expanders - Apply equivalent subjects Search modes - Proximity | 42,487 |
| S20 | (MH "Heart Failure+") | Expanders - Apply equivalent subjects Search modes - Proximity | 51,246 |
| S21 | (MH "Asthma+") OR "Asthma*" | Expanders - Apply equivalent subjects Search modes - Proximity | 51,172 |
| S22 | chronic obstructive pulmonary disease or COPD | Expanders - Apply equivalent subjects Search modes - Proximity | 32,441 |
| S23 | (chronic and obstructive and (airway or respiratory or lung) and disease) | Expanders - Apply equivalent subjects Search modes - Proximity | 15,195 |
| S24 | (MH "Pulmonary Disease, Chronic Obstructive+") | Expanders - Apply equivalent subjects Search modes - Proximity | 23,548 |
| S25 | Interstitial lung disease or ILD | Expanders - Apply equivalent subjects Search modes - Proximity | 6,175 |
| S26 | (MH "Lung Diseases, Interstitial+") | Expanders - Apply equivalent subjects Search modes - Proximity | 9,766 |
| S27 | cardiovascular disease* or CVD | Expanders - Apply equivalent subjects Search modes - Proximity | 117,088 |
| S28 | (MH "Chronic Disease") OR "chronic disease*" | Expanders - Apply equivalent subjects Search modes - Proximity | 98,758 |
| S29 | "long term condition*" | Expanders - Apply equivalent subjects Search modes - Proximity | 30,485 |
| S30 | S15 OR S16 OR S17 OR S18 OR S19 OR S20 OR S21 OR S22 OR S23 OR S24 OR S25 OR S26 OR S27 OR or S28 or S29 | Expanders - Apply equivalent subjects Search modes - Proximity | 821,481 |
| S31 | (MH "Critical Path") | Expanders - Apply equivalent subjects Search modes - Proximity | 6,346 |
| S32 | (MH "Consensus") | Expanders - Apply equivalent subjects Search modes - Proximity | 9,694 |
| S33 | (MH "Practice Guidelines") | Expanders - Apply equivalent subjects Search modes - Proximity | 89,931 |
| S34 | PT (guideline or practice guideline or consensus development conference or consensus development conference) | Expanders - Apply equivalent subjects Search modes - Proximity | 0 |
| S35 | position statement* or policy statement* or practice parameter* or best practice* | Expanders - Apply equivalent subjects Search modes - Proximity | 32,749 |
| S36 | standards or guideline or guidelines | Expanders - Apply equivalent subjects Search modes - Proximity | 662,716 |
| S37 | AB (((practice or treatment* or clinical) NEXT guideline*)) | Expanders - Apply equivalent subjects Search modes - Proximity | 0 |
| S38 | ((practice or treatment* or clinical) NEXT guideline*) | Expanders - Apply equivalent subjects Search modes - Proximity | 0 |
| S39 | TI (CPG or CPGs) | Expanders - Apply equivalent subjects Search modes - Proximity | 444 |
| S40 | TI consensus* | Expanders - Apply equivalent subjects Search modes - Proximity | 12,440 |
| S41 | ((critical or clinical or practice) N2 (path or paths or pathway or pathways or protocol*)) | Expanders - Apply equivalent subjects Search modes - Proximity | 16,479 |
| S42 | TI recommendat* OR AB guideline recommendation* | Expanders - Apply equivalent subjects Search modes - Proximity | 28,025 |
| S43 | (care n2 (standard or path or paths or pathway or pathways or map or maps or plan or plans)) | Expanders - Apply equivalent subjects Search modes - Proximity | 90,033 |
| S44 | (algorithm* n2 (screening or examination or test or tested or testing or assessment* or diagnosis or diagnoses or diagnosed or diagnosing)) | Expanders - Apply equivalent subjects Search modes - Proximity | 3,378 |
| S45 | AU guideline* or standards or consensus* or recommendat* | Expanders - Apply equivalent subjects Search modes - Proximity | 634,292 |
| S46 | CA guideline* or standards or consensus* or recommendat* | Expanders - Apply equivalent subjects Search modes - Proximity | 634,858 |
| S47 | S31 OR S32 OR S33 OR S34 OR S35 OR S36 OR S37 OR S38 OR S39 OR S40 OR S41 OR S42 OR S43 OR S44 OR S45 OR S46 | Expanders - Apply equivalent subjects Search modes - Proximity | 853,069 |
| S48 | S14 AND S30 AND S47 | Expanders - Apply equivalent subjects Search modes - Proximity | 7,514 |
| S49 | EM 2000- | Expanders - Apply equivalent subjects Search modes - Proximity | 6,943,577 |
| S50 | S48 AND S49 | Expanders - Apply equivalent subjects Search modes - Proximity | 7,150 |
| S51 | EM 20220208- | Expanders - Apply equivalent subjects Search modes - Proximity | 887,264 |
| S52 | S48 AND S51 | Expanders - Apply equivalent subjects Search modes - Proximity | 1,127 |

**Search Strategy B:** Rapid Updated Search (July 2025–March 2026), MEDLINE (Ovid), Search Conducted 10 March 2026

1	exp Exercise/	284,047

2	(exercise* or "physical activit*").ti,ab,kw.	561,230

3	1 or 2	659,954

4	exp Heart Failure/	164,403

5	exp Asthma/	151,330

6	exp Pulmonary Disease, Chronic Obstructive/	74,686

7	(heart failure or asthma* or COPD or "chronic obstructive pulmonary disease*").ti,ab,kw.	530,947

8	4 or 5 or 6 or 7	612,031

9	exp Practice Guideline/	36,098

10	exp Guideline/	42,777

11	exp Guideline/	42,777

12	exp Consensus/	24,965

13	(guideline* or standard* or guidance or recommend* or consensus).ti,ab,kw.	3,453,841

14	10 or 11 or 12 or 13	3,466,232

15	3 and 8 and 14	6,927

16	limit 15 to yr="2025 - 2026"	608

17	limit 16 to ed=20250701-20260228	306
