# Supplementary material for: A systematic review of physical activity guidelines for adults with cardio-respiratory diseases: Stepping towards evidence-based recommendations
Source: Clin Rehabil. 2026 Apr 24;40(9):1143–59. doi: 10.1177/02692155261441158 (PMC13525178; doi:10.1177/02692155261441158)
Supplement: Supplementary file 2 [file sj-docx-2-cre-10.1177_02692155261441158.docx]

**Supplementary Material: Summary of precautions or adaptations extracted**

**COPD**

**Exacerbation Management**

- Monitor clinical status and consider modifying or pausing exercise during exacerbations. Adjust exercise intensity in cases of angina, ischaemia, or hemodynamic instability. *(ESSA)*
- Advise individuals with COPD that physical inactivity increases the risk of future exacerbations. *(NICE)*

**Adaptation Strategies**

- Exercise and activity strategies should be tailored to each individual's daily living needs. Plans must include quantifiable, feasible objectives, agreed upon and documented with the patient. *(GesEPOC)*
- Self-management should incorporate exercise education and lifestyle modifications. *(V/DoD, ERS, GOLD)*

**Medication Use**

- In patients with infrequent symptoms, short-acting β2-agonists may reduce symptoms and improve exercise tolerance. *(GesEPOC)*
- Optimizing bronchodilator therapy (including LAMA and LABA) can enhance exercise training benefits by reducing resting and dynamic hyperinflation, supporting better functional outcomes. *(GOLD)*

**Pulmonary Rehabilitation**

- Increasing physical activity focus may lead to more referrals to pulmonary rehabilitation, a cost-effective intervention for COPD. *(NICE)*
- Pulmonary rehabilitation improves dyspnea, health status, and exercise tolerance especially in individuals with moderate to severe COPD. *(COPDX, GOLD)*
- Offer breathing exercises to those with activity-limiting dyspnea, and refer stable patients with ongoing exercise limitations (despite medication) to pulmonary rehabilitation. *(V/DoD)*

**Air Quality**

- Encourage individuals to stay indoors when air quality is poor, as this can significantly affect COPD symptoms and increase the risk of exacerbations. *(BCG)*

**ASTHMA**

**Triggers**

- Avoid exposure to known allergens (both indoor and outdoor), cold air, and pollution. *(GINA)*

**Weather**

- It is recommended to avoid strenuous outdoor physical activities in cold weather, low humidity, or high air pollution. *(SIA)*

**Air Quality**

- Check the air quality index and avoid, where possible, exercising outside when the air pollution or pollen levels (if a trigger) are high. *(NAEPP, GINA, EAACI)*
- Prefer early mornings and green/blue spaces to reduce exposure to pollutants. *(EAACI)*
- Use indoor exercise options during extreme weather conditions or high pollution/pollen periods; HEPA filters and good ventilation are recommended indoors. *(EAACI)*

**Exercise-induced Advice**

- Appropriate precautions should be taken to avoid exercise-induced asthma. *(SIGN)*
- Encourage patients to take an inhaler as needed pre-exercise to reduce exercise-induced symptoms. *(BCG)*
- If exercise provokes asthma, this is an indicator of poor control and should lead to a review of treatment, rather than exercise avoidance. *(NZ-AARF, NAEPP)*
- Reliever medication should always be available to reverse exercise-induced bronchoconstriction should it occur. *(NZ-AARF)*
- If exercise-induced bronchoconstriction occurs, asthmatics should cease exercise or continue with caution. *(AAESS)*
- Exercise only if controller and preventive medications have been administered pre-exercise. *(AAESS)*
- Exercise-induced asthma should be prevented by long-term management with controller drugs and inhalation of beta-2 stimulants before exercise. *(NAEPP, AAESS)*
- Doctors should be consulted before taking medicine for prevention of symptoms during exercise. *(NAEPP)*
- A warm-up period before exercise may reduce exercise-induced bronchoconstriction (EIB) symptoms. If this approach does not control symptoms, the patient is recommended to have maintenance therapy with inhaled corticosteroids. *(SIA, NAEPP, GINA)*
- Include short high-intensity intervals during warm-up to reduce EIB risk. *(EAACI)*
- Use short-acting beta-agonists (SABA) or low-dose ICS prior to activity for managing EIB. *(GINA)*

**Medical and Practical Considerations**

- Medical clearance is required for sedentary individuals or those with cardiovascular, metabolic, or renal history. *(EAACI)*
- Postpone exercise after acute exacerbations until stable. *(EAACI)*
- Adherence to personalised asthma action plans is essential. *(EAACI)*
- Tailor exercise type, intensity, and setting to individual needs, preferences, and asthma control level. *(EAACI)*
- Use tools such as heart rate zones, RPE scales, wearables, or the “talk test” to monitor intensity during exercise. *(EAACI)*

**Masks and Altitude Considerations**

- Consider N95/FFP2/FFP3 masks for PM exposure (assess breathing load). *(EAACI)*
- Use heat-moisture exchange masks or scarves during cold weather to reduce EIB risk. *(EAACI)*
- Altitude therapy may help in severe or poorly controlled asthma by avoiding pollution/allergens. *(EAACI)*

**Heart Failure**

**Exercise Safety**

- Clinical reassessment should be considered when the intensity of exercise is increased. *(ESC1)*
- Ventricular rate control is recommended at rest and during exercise in individuals with both HF and atrial fibrillation. *(CCS)*
- Competitive sports are not recommended in individuals at high risk of exercise-induced adverse events. *(ESC1)*
- Intensive exercise should be avoided in those with severe valvular heart disease, poor left ventricular function, arrhythmogenic cardiomyopathy, or active myocarditis. *(ESC1)*
- Patients receiving anticoagulation therapy or with an implantable cardioverter-defibrillator (ICD) should avoid contact sports or activities involving potential trauma. *(ESC1)*

**Adaptation Strategies**

- Individuals with HF should be supported to integrate physical and recreational activities into all aspects of daily living and care. *(CAG)*
- Multidisciplinary collaboration between healthcare professionals is encouraged to optimise daily physical activity. *(CAG)*
- Motivational and psychological support, along with personalised advice on increasing the amount and intensity of activity, should be considered. *(ESC1)*

**Rehabilitation**

- Supervised, exercise-based cardiac rehabilitation is recommended for all stable individuals to improve exercise capacity, quality of life, and reduce hospital readmissions. *(ESC1, ESC2)*
- Cardiac rehab is especially recommended for people with severe HF, frailty, or comorbidities. *(ESC2)*
- Consider structured exercise referrals and address individual barriers or concerns to promote safe physical activity. *(ESC1, ESC2)*

**High-Intensity Interval Training (HIIT)**

- HIIT programmes may be considered in low-risk individuals seeking to return to high-intensity aerobic or mixed endurance activities. *(ESC1)*
